# Discovery and structural characterisation of new fold type IV-transaminases exemplify the diversity of this enzyme fold

**DOI:** 10.1038/srep38183

**Published:** 2016-12-01

**Authors:** Tea Pavkov-Keller, Gernot A. Strohmeier, Matthias Diepold, Wilco Peeters, Natascha Smeets, Martin Schürmann, Karl Gruber, Helmut Schwab, Kerstin Steiner

**Affiliations:** 1 acib, Austrian Centre of Industrial Biotechnology GmbH, 8010 Graz, Austria; 2 DSM, Ahead R&D - Innovative Synthesis BV, NL-6167 RD Geleen, The Netherlands; 3 Institute of Molecular Biosciences, University of Graz, 8010 Graz, Austria; 4 Institute of Molecular Biotechnology, TU Graz, 8010 Graz, Austria

## Abstract

Transaminases are useful biocatalysts for the production of amino acids and chiral amines as intermediates for a broad range of drugs and fine chemicals. Here, we describe the discovery and characterisation of new transaminases from microorganisms which were enriched in selective media containing (*R*)-amines as sole nitrogen source. While most of the candidate proteins were clearly assigned to known subgroups of the fold IV family of PLP-dependent enzymes by sequence analysis and characterisation of their substrate specificity, some of them did not fit to any of these groups. The structure of one of these enzymes from *Curtobacterium pusillum*, which can convert d-amino acids and various (*R*)-amines with high enantioselectivity, was solved at a resolution of 2.4 Å. It shows significant differences especially in the active site compared to other transaminases of the fold IV family and thus indicates the existence of a new subgroup within this family. Although the discovered transaminases were not able to convert ketones in a reasonable time frame, overall, the enrichment-based approach was successful, as we identified two amine transaminases, which convert (*R*)-amines with high enantioselectivity, and can be used for a kinetic resolution of 1-phenylethylamine and analogues to obtain the (*S*)-amines with *e.e.*s >99%.

Biocatalytic processes have become increasingly important in chemical industry^1^,^2^,^3^. Of particular importance is one property of enzymes - their stereoselectivity - which enables one of the two enantiomers to be preferentially reacted or formed in chemical reactions with chiral or prochiral compounds. Enantiopure chiral amines and amino acids are important building blocks in the synthesis of many pharmaceuticals, fine chemicals and agrochemicals. Within the expanding biocatalytic toolbox for amine synthesis^4^, especially amine transaminases (ATAs) have been studied extensively during the past few years^5^,^6^,^7^. In general, transaminases catalyse the transfer of an amino group from an amino donor to a carbonyl acceptor with pyridoxal 5′-phosphate (PLP) as cofactor. They belong to fold types I and IV of PLP-dependent enzymes. While α-transaminases such as l-branched chain aminotransferases (BCATs) and d-amino acid aminotransferases (D-ATAs) use solely α-amino acids as amino donors, amine transaminases can use amines as donors as well.

In addition to excellent enantioselectivity, high conversions and a broad substrate scope, also high space-time yields, high substrate loadings and process stability are mandatory for an efficient industrial process. Although a lot of effort has already been made to improve and tailor transaminases according to industrial requirements by protein, substrate and reaction engineering^8^,^9^,^10^,^11^,^12^, there is still a clear need for further enhancement of known transaminases or the discovery of new, especially (*R*)-selective transaminases ((*R*)-ATA) exhibiting improved properties. All (*R*)-amine transaminases described in the literature so far are members of the fold type IV PLP-dependent enzyme family^13^,^14^,^15^,^16^,^17^,^18^. This family includes d-amino acid aminotransferases, L-branched chain aminotransferases and 4-amino-4-deoxychorismate lyases (ADCL)^19^.

Methods used for the discovery of novel enzymes can be classified as either activity or sequence based^20^. During activity based enzyme discovery microorganisms are either enriched on selective media containing the substrate of choice or different organisms are screened for their ability to convert the desired substrates. Several amine transaminases have been discovered by applying this method^21^,^22^,^23^,^24^. On the other hand, with an increasing number of genome sequences and protein structures available, database mining in combination with *in silico* prediction of key amino acid residues has become an increasingly successful tool^14^,^25^,^26^,^27^.

Herein, we report the discovery of a new class of fold IV-transaminases, which was discovered during our quest for novel (*R*)-selective transaminases using an enrichment-based approach.

## Results and Discussion

### Identification of genes encoding putative (*R*)-selective transaminases

**Figure 1:**
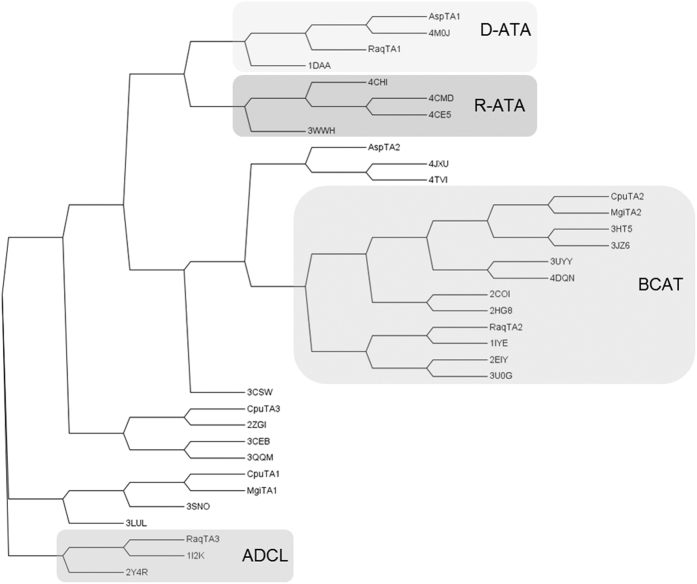
Cladogram of selected fold IV transaminases: the newly discovered transaminases and sequences of fold IV aminotransferases with solved structures. D-ATAs: 1DAA from *Bacillus* sp YM-1^31^, 4M0J from *Burkholderia thailandensis* E264; R-ATAs: 4CE5 from *Aspergillus terreus*^15^, 4CHI from *Aspergillus fumigatus*^18^, 4CMD from *Nectria haematococca*^17^, 3WWH from *Arthrobacter* sp. KNK168^13^; BCATs: 1IYE from *E. coli*^32^, 2EIY from *Thermus thermophilus*, 3HT5 from *Mycobacterium tuberculosis*^47^, 3JZ6 from *Mycobacterium smegmatis*^48^, 3UYY from *Deionococcus radiodurans*^49^, 3U0G from *Burkholderia pseudomallei*, 4DQN from *Streptococcus mutans*^50^, 2COI^36^ and 2HG8^51^ from human; ADCLs: 1I2K from *E. coli*, 2Y4R from *Pseudomonas aeruginosa*^52^, and a few putative aminotransferases: 2ZGI from *Thermus thermophilus* HB8^53^, 3CEB from *Haemophilus somnus*, 3CSW from *Thermotoga maritima*, 3LUL from *Legionella pneumophila*, 3QQM from *Mesorhizobium loti,* 3SNO from *Corynebacterium glutamicum*, 4JXU from *Sinorhizobium meliloti* and 4TVI from *Brucella abortus.* The sequences were aligned using ClustalW2^54^ and the phylogenetic tree was visualised using the programme Archaeopteryx^55^.

Soil samples from various places in the Netherlands and Germany were used for the enrichment of microorganisms, which can use (*R*)-amines such as (*R*)-2-aminobutane, (*R*)-3,3-dimethyl-2-aminobutane, (*R*)-1-phenylethylamine, (*R*)-1-phenylpropylamine, (*R*)-1-aminoindane and (*R*)-1-aminotetralin as sole nitrogen source. Several bacterial and fungal strains were isolated and classified using bacterial or fungal 16 S or D2-LSU rRNA sequences^28^. The genomes of the four most active bacterial strains, which were enriched on different amines - *Rahnella aquatilis* ((*R*)-1-aminotetralin)*, Microbacterium ginsengisoli* ((*R*)-1-phenylpropylamine)*, Curtobacterium pusillum* ((*R*)-1-aminoindane), and *Achromobacter spanius* ((*R*)-1-phenylethylamine) - were sequenced. The genome sequences were used for sequence based similarity searches against known (*R*)-amine transaminase sequences (either from public databases or from an in-house platform) with confirmed (*R*)-ATA activity. Several candidates with different degrees of similarity were identified (Table S1), whereby the overall sequence similarity was rather low (highest similarity of only 26.9% to AT-ωTA from *Aspergillus terreus*). However, we have found before that an enzyme with only 34% sequence identity to AT-ωTA showed (*R*)-ATA activity (unpublished data), and overall the sequences in this subfamily are diverse. Comparison of the sequences to those of fold IV transaminases with known structures (Fig. 1) revealed that *Asp*TA1 and *Raq*TA1 are most similar to D-ATAs, *Cpu*TA2, *Mgi*TA2 and *Raq*TA2 to BCATs and *Raq*TA3 to ADCLs. None of the protein sequences were aligned in the same subgroup as currently known (*R*)-ATAs. *Asp*TA2, *Cpu*TA1, *Cpu*TA3 and *Mgi*TA1 did not fit to any of the characterised subgroups. Interestingly, several biochemically uncharacterised transaminases, which were included in the comparison as their structure was available in the PDB, form additional subgroups in the cladogram (Fig. 1).

Bornscheuer and his group identified sequence motifs, which are characteristic for each subgroup of the fold type IV PLP dependent enzyme family^14^. Based on that, the protein sequences were analysed for the presence of these conserved amino acid motifs (Table S2). Again, none of the candidate proteins was clearly predicted to be an (*R*)-amine transaminase. *Asp*TA1 and *Raq*TA1 share 45% sequence identity and are predicted to be D-ATAs. They contain all of the conserved amino acids for D-ATAs, but contain also some of the predicted amino acids of (*R*)-ATAs, which the two subfamilies share. *Cpu*TA1 and *Mgi*TA1 share 49% sequence identity and contain conserved amino acids of (*R*)-ATA and ADCLs, thus cannot be explicitly assigned to any of the subgroups. *Cpu*TA2 and *Mgi*TA2 share 71% sequence identity and are predicted to be BCATs. Also *Asp*TA2 and *Raq*TA2 show highest similarity to BCATs, although their similarity to each other and to *Cpu*TA2 and *Mgi*TA2 is only between 20 and 30%. *Cpu*TA2, *Mgi*TA2 and *Raq*TA2 contain all conserved amino acids predicted for BCATs, while *Asp*TA2 is different at two positions (Table S2). *Raq*TA3 exhibits a high similarity to ADCLs. *Cpu*TA3 is predicted to belong to the aminotransferase type IV fold family, but it is not very similar to any of the other newly identified sequences or the already characterised (*R*)-ATAs. Moreover, it does not comprise any of the conserved amino acids described for the other members of this family.

### Initial characterisation of the transaminases

To further analyse the activity of the candidate proteins, they were heterologously expressed in *E. coli*. All proteins except *Asp*TA1 and *Cpu*TA3 displayed a high expression level. Most of the target proteins were highly soluble, the rest was partially soluble. *Asp*TA1 was expressed with significantly lower yield as soluble protein, and *Cpu*TA3 was almost insoluble. On SDS-PA gels all protein bands were displayed at the expected molecular size (Figure S1).

In a standard spectrophotometrical activity assay for amine transaminases using (*R*)-1-phenylethylamine or (*R*)-1-aminotetralin and pyruvate as substrates, only *Cpu*TA1 and *Mgi*TA1 were active (Table 1).

**Figure 2:**
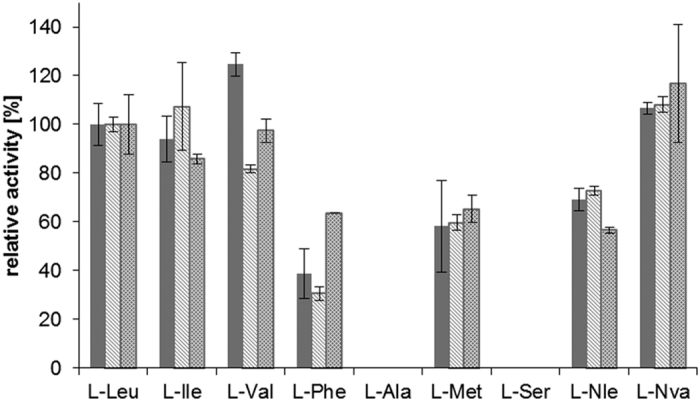
Substrate specificity of several putative branched chain amino acid transaminases. Reaction conditions: 10 mM α-ketoglutaric acid, 5 mM l-amino acid, 0.1 mM PLP, 0.5 mM NAD^+^, 1 U GDH (l-glutamate dehydrogenase) in 50 mM KPi, pH 8, with cell-free *E. coli* lysate containing different TAs (0.125 mg mL^−1^ total lysate protein), following the formation of NADH at 340 nm and 30 °C. The reactions were performed in triplicate. The relative activities were compared to the activity with l-leucine (Table 1). Solid grey: *Cpu*TA2, light grey: *Mgi*TA2, darker grey dashed: *Raq*TA2. l-Nle = l-norleucine, l-Nva = l-norvaline.

*Asp*TA1, *Cpu*TA1, *Mgi*TA1 and *Raq*TA1 showed activities in an LDH coupled d-amino acid transaminase assay using α-ketoglutaric acid and d-alanine as substrates (Table 1). l-alanine was not accepted as substrate. Unsurprisingly, the three candidates (*Cpu*TA2, *Mgi*TA2, *Raq*TA2), which were clearly predicted to be L-branched chain aminotransferases, were active towards various l-amino acids (Table 1 and Fig. 2), confirming the prediction. *Asp*TA2, *Cpu*TA3 and *Raq*TA3 were inactive in all assays with all tested substrates.

**Figure 3:**
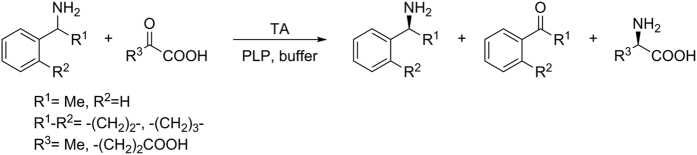
Transaminase-catalysed reactions of *rac*-1-phenylethylamine, *rac*-1-aminotetralin and *rac*-1-aminoindane with α-keto acids.

The reaction products of several amination reactions were also analysed by rp-HPLC to establish the substrate scope and stereopreference of the transformations (Tables 2 and 3). In these reactions all active enzymes exclusively showed (*R*)-selectivity (Fig. 3).

As already observed in the online photometric assay described above (Table 1), *Cpu*TA1 and *Mgi*TA1 were clearly active with several racemic amine donors, reaching up to 50% conversion in the presence of excess pyruvate, which corresponds to a full conversion of the (*R*)-enantiomers (Table 2). All (*S*)-enantiomers were left unconverted without any exception and this property would qualify *Cpu*TA1 and *Mgi*TA1 for kinetic resolutions of racemic amines. After substantially longer reaction times than in the photometric assay, also *Raq*TA3 showed some conversion with all three amines (Table 2).

**Figure 4:**
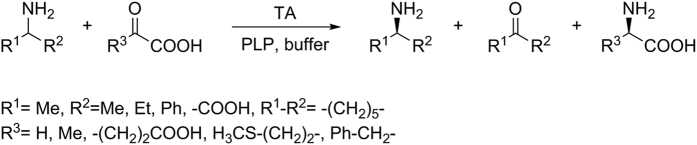
Transaminase-catalysed conversions of α-keto acids to α-amino acids.

*Cpu*TA1, *Mgi*TA1 and *Raq*TA3 were also active in amination reactions of structurally more demanding α-keto acids (Fig. 4). They can thereby transfer the amino group from primary amines bearing an aromatic substituent in close proximity. In sharp contrast, the enzymes provided only low conversions in the presence of excess aliphatic amino donors like propan-2-amine or butan-2-amine (Table 3). The highest conversions, especially with the larger substrates in this study, were obtained using dl-alanine as donor. *Cpu*TA1 was superior to *Mgi*TA1 in the case of the sterically more demanding phenylpyruvate as substrate. As cell-free lysates were used for the reactions, typically complete racemisation of the initially formed d-alanine was found. This observation can be explained by the action of an amino acid racemase present in *E. coli*. In case of the other amino acid products, this competing activity is much weaker and the amount of racemisation almost negligible. Whereas 1-phenylethylamine served as amino donor, the corresponding ketone 1-phenylethanone remained completely unconverted as substrate in the amination reaction mode. Moreover, none of the new TAs showed activity in amination reactions with neither alanine nor 1-phenylethylamine when the following ketones were used as amine acceptors: acetone, 4-methyl-2-pentanone, 1-phenylethanone, 1-phenylpropanone, 1-phenylbutanone, 4-phenylbutan-2-one, 1-tetralone, or 1-indanone (data not shown).

### Characterisation of *Cpu*TA1 and *Mgi*TA1

**Figure 5:**
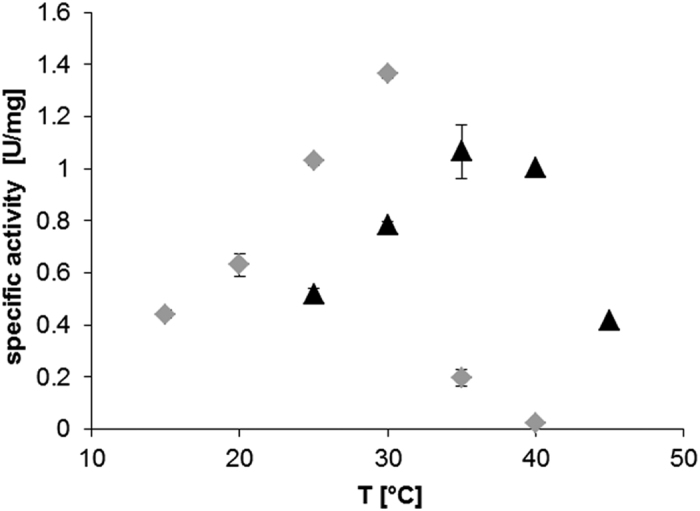
Temperature dependence of specific activities calculated from photometric activity assays using 0.15 mg mL^−1^ of purified *Cpu*TA1 (**A**) or 0.1 mg mL^−1^ of *Mgi*TA1 (**B**) in 50 mM KPi, pH 8, at various T. The reaction mixtures (5 mM pyruvate, 5 mM (*R*)-1-aminotetralin, 0.1 mM PLP in buffer) were incubated for 5 minutes at the respective temperature before addition of pyruvate to start the reaction. The measurements were done in triplicate. Grey diamonds: *Cpu*TA1, black triangles: *Mgi*TA1.

Out of the ten target enzymes, *Cpu*TA1 and *Mgi*TA1 showed the highest activities as amine transaminases and were therefore characterised in more detail. Both enzymes were recloned to introduce a C-terminal His-Tag and were purified by affinity chromatography in high yield and purity (data not shown). The activity of the purified enzymes was investigated under different reaction conditions (buffer, pH, T) in a photometric activity assay. In general, both enzymes were not active at a pH lower than 7.0, and the activities decreased also significantly at pH higher than 10. The optimal activity for *Cpu*TA1 was observed at pH 8 with potassium phosphate buffer (KPi). *Mgi*TA1 showed a similar pH profile as *Cpu*TA1, but was generally more active in Tris/HCl than in KPi buffer (data not shown). The activity of *Cpu*TA1 was highest at 30 °C, but was drastically reduced at temperatures higher than 30 °C (Fig. 5). *Mgi*TA1 was clearly more stable at higher temperatures (up to 40 °C) under the same assay conditions.

### Structural analysis of *Cpu*TA1

#### The overall structure of CpuTA1

**Figure 6:**
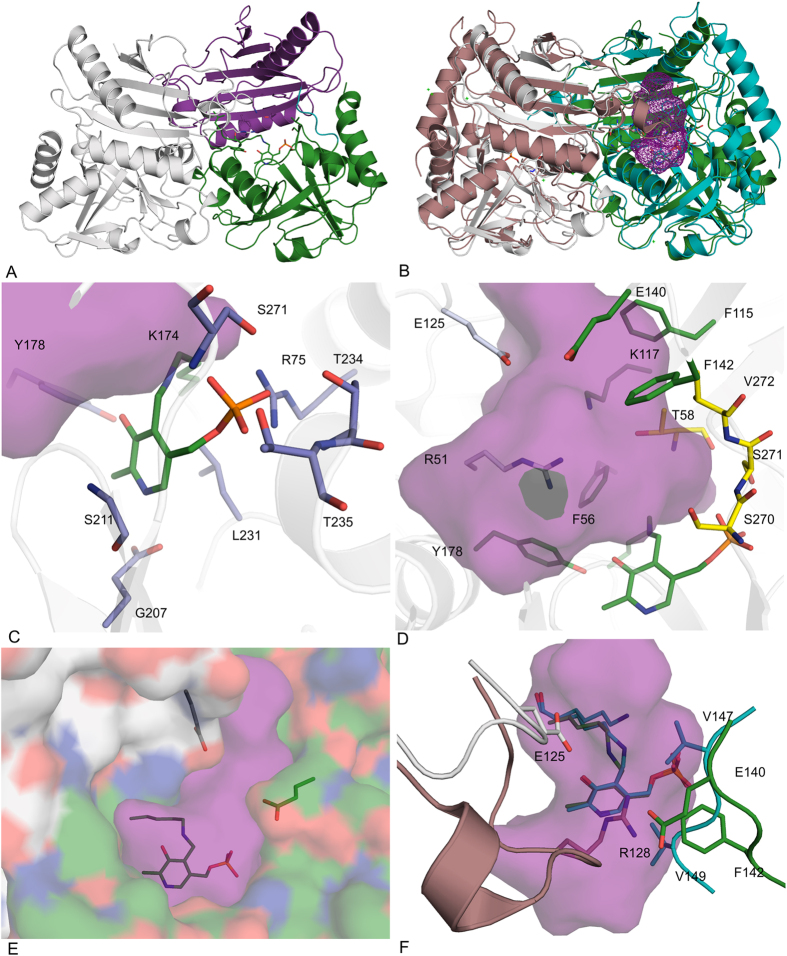
Crystal structure of *Cpu*TA1. (**A**) Overview of the *Cpu*TA1 dimer (chain A first domain in magenta, second domain in green, chain B in grey); (**B**) Overview of a superposition of the *Cpu*TA1 dimer (chain A in green, chain B in grey) with the AT-ωTA dimer (Pdb-code: 4CE5, chain A in turquoise, chain B in brown) with the active site cavity of *Cpu*TA1 depicted as magenta mesh; (**C**) PLP binding amino acids (blue); (**D**) active site amino acids (small binding pocket in yellow, large binding pocket in green); (**E**) entrance tunnel; (**F**) variable loops of *Cpu*TA1 compared to AT-ωTA. The cavity analysis was calculated by Casox^56^. The figures were prepared using the program PyMOL (Schrodinger Inc.).

The crystal structure of *Cpu*TA1 was determined by X-ray crystallography at a resolution of 2.4 Å. The final model contains 587 amino-acid residues (chain A and B), two pyridoxal-5′-phosphate (PLP), one 3-amino benzoic acid (3-ABA) and 327 water molecules. Some residues with poor electron density at the N- and C-terminus were excluded from the final structure (chain A: M1, A16-R25 and H309-H313; chain B: M1-T2, A16-A27 and D305-H313). Crystallographic data processing and structure refinement statistics are presented in Table 4. The asymmetric unit contains two polypeptide chains, which form a stable dimer with a contact surface of 5580 Å^2^ (PDBePISA^29^) (Fig. 6A). The interface contacts are mostly van der Waals interactions and hydrogen bonds. One chain divides into two domains (N-terminus to E138 and Q144 to C-terminus), which are separated by an interdomain loop. *Cpu*TA1 exhibits the typical aminotransferase type IV fold (InterPro: IPR001544, Pfam: Pf01063). A structural similarity search using the DALI server^30^ did not result in a closely matching hit. However, the closest homologous structures with confirmed function are D-amino acid aminotransferases (e.g. PDB-code: 1DAA, a D-ATA from *Bacillus* sp. (strain YM-1)^31^, Z-score: 31.2, rmsd 2.3 Å, seq id: 20%), but also branched chain amino acid aminotransferases (e.g. PDB-code: 1IYE, a BCAT from *E. coli*^32^, Z-score: 30.3, rmsd 2.2 Å, seq id: 19), and (*R*)-selective transaminases (e.g. PDB-code: 4CE5 from *Aspergillus terreus*^15^, Z-score: 29.0, rmsd 2.2 Å, seq id: 22%). (*R*)-selective amine transaminases contain an N-terminal α-helix spanning the initial 20 amino acids^15^,^18^, which is not observed in any of the other fold IV subfamilies. This helix is also not present in *Cpu*TA1 (Fig. 6B).

#### Active site

The electron density for the PLP cofactor is well defined and the covalent imino bond to the active site Lys174 residue is clearly visible. The PLP binding site and the amino acids involved are highly similar to other fold IV enzymes (Fig. 6C). The phosphate group is interacting through a network of hydrogen bonds to the side chains of Arg75, Thr234, Thr235 and Ser271, the backbone amides of Thr234, Thr235 and Ser271, and two water molecules. The OH-group of PLP is in close vicinity of the OH-group of Tyr178. The pyridine ring of PLP is sandwiched between the side chain of Leu231 and the backbone of Ser211. Glu207 forms a hydrogen bond between its side chain carboxyl group and the nitrogen atom of the pyridine ring.

Amino acids of both chains contribute to the formation of the active site, as observed for other members of fold IV enzymes. The two active sites are located at the interface of the two polypeptide chains with the main portion located in one chain, and *vice versa*. The entrance to the active site is situated at the dimer interface as well. The active site is surrounded by the residues R51*, F56, T58, F115, K117, E125, E140, F142, Y178, S270, S271, and V272 (*indicates that the residues belongs to the other chain) (Fig. 6D). These amino acids are building two binding pockets, whereby the small binding pocket is lined by T58, S270, S271 and V272 and the large binding pocket by R51*, F56, K115, E125*, E140, F142 and Y178. The large binding pocket is open to the protein surface forming an entrance tunnel, whereby E125* together with E142 seems to be the bottleneck of the entrance tunnel (Fig. 6E). Overall, the structure of *Cpu*TA1 confirms that except for the PLP binding amino acids hardly any of the active site amino acids is conserved compared to the characterised fold IV subgroups. The enantiopreference of BCATs and D-ATAs is determined by the localisation of the α-carboxyl group in relation to PLP^32^,^33^. In BCATs the α-carboxylate is located on the phosphate group side of the cofactor interacting with the hydroxy group of a tyrosine, which is polarised by an arginine^32^. These residues are not conserved in *Cpu*TA1 and no tyrosine or positively charged amino acids are in close distance. In D-ATAs the α-carboxylate is bound in the binding pocket above the O3’ of PLP and the oxygen of the carboxyl group interacts with a positively charged arginine and in addition with a histidine and a tyrosine residue^34^. Although these residues, which are part of a flexible loop, are not conserved in *Cpu*TA1, R51 from an adjacent loop of chain B (corresponding to H55 in AT-ωTA) and K117 (corresponding to E117 in AT-ωTA) from chain A are clearly pointing towards the active site and a potentially bound α-carboxylate. Thus, the structural data support the experimentally determined enantiopreference (D, *R*) of *Cpu*TA1. Interestingly, a flexible loop (e.g. aa K125-I135 in AT-ωTA), which is present in (*R*)-ATAs^13^,^15^,^17^,^35^ as part of the active site (the loop of chain B as part of the active site of chain A and *vice versa* containing an arginine residue, which is discussed to play a role in dual substrate recognition of (*R*)-ATAs^13^,^15^,^35^) is significantly shorter in *Cpu*TA1 (aa E125-G128) and does not contain any positively charged amino acid. Thus, the loop is located further away from the PLP binding site creating more space in the active site (Fig. 6F). This loop changes its conformation from a closed to an open form upon binding of the inhibitor gabaculin in the (*R*)-ATA of *A. fumigatus*^35^ thereby moving R126 out of the active site, and due to high B-factors of the amino acid residues the loop is also expected to be flexible in AT-ωTA of *A. terreus*^15^. Moreover, an altered conformation of the respective loop in ATA-117-Rd11 variant compared to its wildtype might be one of the reasons for its ability to accommodate the pro-sitagliptin ketone^13^. While the exact position of R138 in the loop of ATA-117-Rd11 is not conserved compared to other published (*R*)-ATA structures, the guanidinium groups of all loop arginines localise in a similar position. A similar loop as in (*R*)-ATAs is present but not conserved in most members of fold type IV transaminases. Compared to the structure of (*R*)-ATAs, e.g. of *A. terreus* and *A. fumigatus,* the active site of *Cpu*TA1 is further slightly opened up as the interdomain loop E138-Q144 is located further away from the PLP (corresponding to loop P144-A150 containing I146 and V148 which were predicted to be part of the active site of the (*R*)-ATA of *A. fumigatus*^18^. In contrast, the respective loop in D-ATAs is located significantly further away, which is in correspondence with the prediction that the positions of the small and the large binding pocket in relation to the O3′ and the phosphate group of PLP are complementary in (*R*)-ATA and D-ATAs. Interestingly, in several BCATs this interdomain loop is disordered in structures of unliganded forms. It appears to be very flexible, changes conformation upon substrate binding and differs between BCATs of different origin and even between isoenzymes^32^,^36^.

## Conclusions

In the last decade, the definition of transaminases became more complex. In addition to α-transaminases, which catalyse the conversion of α-amino acids to α-keto acids and vice versa, and β and ω-transaminases, which transfer amino groups which are more distant from the carboxylic group, also the group of amine transaminases was discovered and defined. Amine transaminases can convert amines independently of the presence of a carboxylic group, albeit still able to convert e.g. amino acids as well, depending on their substrate scope^14^,^27^. They are especially interesting in biocatalysis for their ability to catalyse also the reverse reaction converting ketones to amines. During our quest for new (*R*)-selective amine transaminases, we isolated ten candidate enzymes, of which two were clearly identified as D-ATAs and three as BCATs. One showed high sequence similarity to ADCLs. For four candidates, however, the prediction and biochemical data were less clear. This once more highlights the difficulty of predicting function from sequence, which is only possible, if enough biochemical data are already available^27^, and consequently, the need to close the gap between sequence and functional information. In contrast to exclusive amino acid transaminases such as D-ATAs and BCATs, *Cpu*TA1 and *Mgi*TA1 are in addition able to convert amines to ketones, which would categorise them in the subclass of amine transaminases. However, those enzymes are commonly able to catalyse also the reverse reaction in reasonable reaction times. In the case of *Cpu*TA1 and *Mgi*TA1, a reverse reaction was not detectable during the investigated time scale. Considering this and based on sequence and structural analysis, which revealed significant differences especially also in the active site, we propose that *Cpu*TA1 and *Mgi*TA1, might form a new subgroup in the fold IV family of PLP-dependent enzymes. Moreover, the fact that there are several structures of proteins with uncharacterised function and similarity to the fold type IV family of PLP-dependent enzymes deposited in the protein database, which differ in their active site amino acids and cannot be clearly assigned to one of the known fold type IV subgroups, indicates that the fold type IV family is a lot more diverse and many more activities and substrate specificities are still to be discovered.

Furthermore the results also show that the directed evolution paradigm “you get what you screen for” can also apply for a bioprospecting approach of classical enrichment combined with Bio-IT inspection of the obtained genome sequence, as presented herein. The strains were enriched for deamination of (*R*)-amines and indeed contained transaminases capable of catalysing such reactions. No enzyme catalysing the conversion of both, amines and ketones, in a reasonable time frame was identified from the four investigated bacterial strains. Most likely because the amination of ketones was no feature of the enrichment selection pressure and, in retrospect, an enrichment assay in the amination direction might have been more difficult to design and perform, but would probably have delivered the desired conversion of ketones to (*R*)-amines.

## Methods

### General Information

All chemicals were purchased from Sigma-Aldrich (St. Louis, MO, USA) or Carl Roth GmbH (Karlsruhe, Germany), if not stated otherwise. Materials for molecular biology were obtained from Thermo Fisher Scientific (Waltham, MA, USA), if not specifically mentioned. For enrichment of microorganisms selective enrichment medium (SEM) consisting of 10 g/L Difco Yeast Carbon Base (YCB, BD, Sparks, MD, USA), 55 mM glycerol (Merck, Darmstadt, Germany), 10 mM pyruvic acid (Sigma-Aldrich, Steinheim, Germany), 5 mM (*R*)-amine substrate was used and for solid enrichment medium it contained also 15 g/L Difco Agar Noble (BD, Le Pont de Claix, France). *E. coli* TOP10F‘ (Invitrogen/LifeTechnologies, Carlsbad, CA, USA) and *E. coli* BL21-Gold(DE3) (Stratagene) were used for protein expression.

### Enrichment of microorganisms on (*R*)-amines as nitrogen source

Soil samples from various places in the Netherlands and Germany were suspended in 100 mM potassium phosphate (KPi) buffer, pH 7.0, and incubated with shaking at 180 rpm and 28 °C for 1 h. The suspensions were filtered through filter paper (Whatman/GE Healthcare, Uppsala, Sweden). Hundred μL of filtrate was used for inoculation of 10 mL SEM (in 100 mL shaking flasks) containing one of the six (*R*)-amines selected from the group (*R*)-2-aminobutane, (*R*)-3,3-dimethyl-2-aminobutane, (*R*)-1-phenylethylamine, (*R*)-1-phenylpropylamine, (*R*)-1-aminoindane and (*R*)-1-aminotetralin. The flasks were incubated with shaking at 180 rpm and 28 °C. When microbial growth was obtained, 100 μL of this culture medium was used to inoculate SEM containing 5 mM of the same (*R*)-amine. After two such passages 100 μL of a 1:100 dilution of the last culture medium was plated on SEM agar plates containing 5 mM of the corresponding (*R*)-amine. Additionally, approximately 10 μL of the undiluted culture was streaked out on SEM agar plates containing 5 mM of the corresponding (*R*)-amine. The inoculated agar plates were incubated at 28 °C until growth was observed. Colonies with different morphology were restreaked on fresh SEM plates to separate different microbial species. The re-streaking was continued until pure cultures with uniform morphologies were obtained after incubation at 28 °C and storage at 4 °C. Selected microorganisms were sent for MicroSEQ^®^ (Applied Biosystems/LifeTechnologies, Carlsbad, CA, USA) Identification to BaseClear (Leiden, The Netherlands). The bacterial or fungal 16 S or D2-LSU rRNA sequences obtained were compared to the validated MicroSEQ^®^ sequence database (at BaseClear, Leiden, The Netherlands) and in case no identity above 99% was obtained compared to the non-redundant (nr) nucleotide database using the BlastN algorithm on the NCBI Blast homepage (http://www.ncbi.nlm.nih.gov/BLAST/).

### Isolation of DNA and cloning procedures

From four strains [*Rahnella aquatilis* 3 Kb (*Raq*), *Microbacterium ginsengisoli* 1A1 DSM 23784 (*Mgi*), *Curtobacterium pusillum* 5BaB DSM 23787 (*Cpu*), and *Achromobacter spanius* 6I DSM 23791 (*Asp*)], which were grown on selective media as described above, genomic DNA was isolated with the Easy-DNA kit (Invitrogen/LifeTechnologies) according to the manufacturer’s manual and finally eluted with 10 mM Tris/HCl, 1 mM EDTA, pH 8.0, (TE) buffer. The quality of the DNA was monitored spectrophotometrically as well as by digest with *Bsp*143I or *Bam*HI followed by agarose gel electrophoresis (data not shown). The isolated genomic DNA samples from *R. aquatilis, M. ginsengisoli, C. pusillum*, and *A. spanius* were used for genome sequencing at IIT Biotech GmbH in Bielefeld, Germany. The sequence data were uploaded to an in-house server at the Institute of Knowledge Discovery at TU Graz and sequence similarity searches were conducted using blastn and tblastn programmes submitting known (*R*)-transaminase sequences and hits found in one of the genomes. Several hits with different degrees of similarity were identified and chosen for subsequent amplification by standard PCR using Phusion High Fidelity DNA polymerase (Thermo Scientific) (primers see Table S3). Three of the genes could not be amplified by PCR (even after optimisation of the reaction conditions) and one gene (*Raq*TA2) contained the motif of the *Nde*I and *Hind*III restriction sites in its native gene sequence and thus *Asp*TA1, *Cpu*TA1, *Cpu*TA3 and *Raq*TA2 were ordered codon-optimised for expression in *E. coli* from Geneart/Life Technologies (Regensburg, Germany). All genes were cloned via *Nde*I/*Hin*dIII into pMS470Δ8^37^. The correctness of the inserts was confirmed by sequencing (LGC Genomics, Berlin, Germany). The two genes of *Cpu*TA1 and *Mgi*TA1 were subcloned into pET28a expression vector for the fusion of a C-terminal His-Tag by PCR amplification and digest with the restriction enzymes *Nco*I and *Xho*I.

### Protein expression and purification

*E. coli* TOP10F’ cells harbouring pMS470-constructs or *E. coli* BL21-Gold(DE3) cells harbouring pET28a-constructs were grown in LB medium supplemented with ampicillin (Amp, final conc 100 μg mL^−1^) or kanamycin (Kan, final conc 50 μg mL^−1^). For the main cultures, the pre-cultures were diluted to an attenuance at 600 nm of ~0.1, and grown in baffled flasks at 37 °C and 120 rpm. Protein expression was induced with 0.5 mM IPTG at an attenuance at 600 nm of ~0.6–0.8 and expression was performed at 25 °C for 20 h. The cells were harvested at 4,000 × g for 15 min, and resuspended in cold buffer (pMS470 constructs: 50 mM KPi buffer, pH 7.5, containing 0.1 mM PLP, or pET28a-constructs: buffer A, 20 mM sodium phosphate buffer, pH 7.4, 0.1 mM PLP, 0.5 M NaCl and 10 mM imidazole), disrupted by sonication (Branson Sonifier S-250; 6 min, 80% duty cycle, 70% output) and centrifuged at 50,000 × g for 1 h. The cleared lysates were filtered through 0.45 μm syringe filters and if necessary concentrated using Vivaspin 20 Centrifugal Filter Units (10,000 Da molecular-weight cut-off; Sartorius, Göttingen, Germany). The protein concentration of the lysate was established by Bradford protein assay. The expression and solubility of the proteins were analysed by SDS-PAGE (NuPAGE Bis-Tris PreCast Gels/Life Technologies). For purification of the His-tagged proteins the filtered cell-free lysates were incubated with Ni Sepharose^TM^ 6 Fast Flow resin (GE Healthcare) for 15 min. The Ni Sepharose^TM^ resin was then filled into empty PD-10 columns (GE Healthcare). After removal of impurities with buffer A containing 30 mM imidazole, the target proteins were eluted with 300 mM imidazole in buffer A. Fractions were analysed by SDS-PAGE, pooled, concentrated and desalted on PD-10 desalting columns (GE Healthcare) into 50 mM KPi buffer, pH 7.5, for biochemical characterisation or 20 mM Tris/HCl, 200 mM NaCl, pH 7.5, for protein crystallisation studies. Concentrations of purified proteins were determined with a Nanodrop spectrophotometer (model 2000c, Peqlab, Erlangen, Germany), using an absorbance of 10.06 for a 1% solution (10 g L^−1^) at 280 nm, calculated based on the amino acid sequence using Protparam^38^. The samples were frozen until further use.

### Spectrophotometric activity assays

#### (R)-amine transaminase assay

The activities of the enzymes were determined using a standard photometric assay^39^ at 25 °C containing 0.25 mg mL^−1^ of total lysate protein, 0.1 mM PLP, 5 mM (*R*)-1-phenylethylamine (PEA), or (*R*)-1-aminotetralin and 5 mM pyruvate in 50 mM KPi, pH 7.5, total volume: 1 mL. The increase of the respective ketones was measured at 300 nm (extinction coefficient of 1-phenylethanone = 0.28 mM^−1^ cm^−1^, extinction coefficient of 1-tetralone = 1.5 mM^−1^ cm^−1^). KPi at pH 7.5 or 8.0 was chosen as buffer for all screenings as transaminases are generally active in this buffer at these pHs, and thus potential candidates should be active as well, even if not all of them might display their highest activity under these conditions.

For a detailed characterisation of *Cpu*TA1 and *Mgi*TA1 purified protein (0.05–0.25 mg mL^−1^) was used. Different amines, (*R*)-1-phenylethylamine, benzylamine, (*R*)-1-phenylpropylamine, (*R*)-1-aminotetralin, and (*R*)-1-aminoindane were tested.

For the determination of the optimal pH and buffer conditions, different buffers were used at a standard concentration of 50 mM: KPi buffer, pH 6.0, 6.5, 7.0, 7.5 and 8.0; Tris/HCl, pH 7.0, 7.5, 8.0, 8.5 and 9.0; Hepes, pH 7.5 and 8.0; glycine, pH 8.5, 9.0, 9.5; carbonate pH 9.2, 9.5 and 10.1. The optimal reaction temperature was ascertained between 15° and 45 °C in 50 mM KPi, pH 8. The reaction mixtures (without pyruvate) were preincubated in the photometer for five min at the respective temperature to ensure constant conditions before starting the reaction by the addition of pyruvate.

#### D-ATA-assay

An LDH (lactate dehydrogenase from rabbit, Sigma) coupled activity assay was performed in a photometer at 25 °C at 340 nm following the depletion of NADH (extinction coefficient of NADH = 6.22 mM^−1^ cm^−1^). One mL of reaction volume contained 5 mM α-ketoglutaric acid, 5 mM D- or L-alanine, 0.5 mM NADH, 40 mU LDH in 50 mM KPi, pH 7.5, and cell-free lysate (0.05–0.0025 mg mL^−1^ total lysate protein). *E. coli* lysate without heterologous transaminase was used as negative control and the obtained slope was subtracted to correct for nonspecific oxidation of NADH.

#### BCAT-assay

A GDH (glutamate dehydrogenase from bovine, Sigma) coupled activity assay^40^ was performed in a photometer at 30 °C at 340 nm following the formation of NADH. One mL of reaction volume contained 10 mM α-ketoglutaric acid, 5 mM L-amino acids (L-leucine, L-isoleucine, L-valine, L-phenylalanine, L-alanine, L-methionine, L-serine, L-norleucine, L-norvaline). 0.1 mM PLP, 0.5 mM NAD^+^, 1 U GDH in 50 mM KPi, pH 8, with cell-free lysate (0.125 mg mL^−1^ total lysate protein).

### General procedure for reactions of amines with α-keto acids

As a representative example, 20 mM *rac*-1-phenylethylamine (250 μL, contains 100 mM KPi, pH 7.5 adjusted with *ortho*-phosphoric acid), 100 mM sodium pyruvate (100 μL), 10 mM PLP (5 μL) and 100 mM KPi, pH 7.5 (145-X μL) were mixed in 1.5 mL reaction tubes and incubated at 30 °C while shaking at 500 rpm. After 10 min, the reaction was started by the addition of lysate (X μL containing 1.0 mg of total protein) and the shaking speed maintained at 800 rpm.

#### Analysis of reactions

An aliquot of the reaction solution (10 μL) was mixed with 10 mM Marfey’s reagent *N*_α_-(2,4-dinitro-5-fluorophenyl)-l-alaninamide in acetonitrile (25 μL, 2.5 eq.) and 1.0 M NaHCO_3_ (10 μL) in an 1.5 mL reaction tube and heated under shaking at 50 °C for 2 h. After cooling, methanol (10 μL) and 4 M HCl (3 μL) were added. Following centrifugation (2 min at 15700 × g), the derivatised sample was analysed by rp-HPLC at a detection wavelength of 340 nm. For HPLC analysis conditions refer to Table S4-5. For higher concentrated amine solutions, lower amounts of samples were used under otherwise identical conditions.

### Crystal structure determination

Screening for crystallisation conditions was performed with an Oryx8 robot (Douglas Instruments) using Morpheus Screen MD 1-46, JCSG+ MD1-37 (Molecular Dimensions) and Index HT HR2-144 (Hampton Research) by the sitting drop vapour-diffusion method in 96-well plates. A stock solution of *Cpu*TA1 at 12 mg mL^−1^ (10 mM Tris/HCl, pH 8) and 5 mM pyridoxal 5′-phosphate hydrate (Sigma Aldrich) was used for all crystallisation experiments. Additional co-crystallisation screens with the inhibitor 3-amino benzoic acid (3-ABA) (10 mM) were setup. Drops of 1 μL were pipetted for screens and optimisations with an 1:1 ratio of protein and screening solution. The crystallisation plates were incubated at 289 K.

Initial crystals of *Cpu*TA1 were obtained in several crystallisation conditions and diffracted to 4 Å resolution at best. Micro-seeding experiments^41^ were set up using those initial crystals of *Cpu*TA1 (Morpheus Screen #13) as seeding stock (1:1000 diluted). In these trials a new crystal form appeared in 3-ABA co-crystallisation screens after two weeks in Index condition 27 [2.4 M sodium malonate pH 7.0] using 10% of microseeds. Data collection was performed on the synchrotron beamlines (Elettra, Trieste, Italy and EMBL, Grenoble, France) at 100 K without additional cryoprotectant. The data sets were processed and scaled using the XDS program package^42^. Molecular replacement was performed with Phaser^43^ using the structure of a putative aminotransferase (NCgl2491) from *Corynebacterium glutamicum* ATCC 13032 (PDB 3SNO, Joint Center for Structural Genomics) as search template. The web service “ARP/wARP: Crystallographic Macromolecular Model Building Version 7.5” was used for initial model building^44^. The resulting model was manually completed in Coot^45^ and refined using the PHENIX software suite^46^. Data collection and processing statistics are summarised in Table 4.

## Additional Information

**Accession codes:** Nucleotide sequence data are available in the DDBJ/EMBL/GenBank databases under the accession numbers KX160484-KX160493. Coordinates and structure factors have been deposited with the Protein Data Bank under accession code 5K3W.

**How to cite this article**: Pavkov-Keller, T. *et al*. Discovery and structural characterisation of new fold type IV-transaminases exemplify the diversity of this enzyme fold. *Sci. Rep.* **6**, 38183; doi: 10.1038/srep38183 (2016).

**Publisher's note:** Springer Nature remains neutral with regard to jurisdictional claims in published maps and institutional affiliations.

## Supplementary Material

Supplementary Material

## Figures and Tables

**Table 1:** Transaminase activities.

Protein	R-ATA activity^a,b^ [U mg^−1^ lysate]	D-ATA activity^c^ [U mg^−1^ lysate]	BCAT activity^d^ [U mg^−1^ lysate]
*Asp*TA1	n.d.	0.13 ± 0.03	n.d.
*Asp*TA2	n.d.	n.d.	n.d.
*Cpu*TA1	0.21 ± 0.00^a^ and 0.29 ± 0.01^b^	0.26 ± 0.00	n.d.
*Cpu*TA2	n.d.	n.d.	0.03 ± 0.00
*Cpu*TA3	n.d.	n.d.	n.d.
*Mgi*TA1	0.16 ± 0.01^a^ and 0.28 ± 0.00^b^	0.03 ± 0.01	n.d.
*Mgi*TA2	n.d.	n.d.	0.08 ± 0.00
*Raq*TA1	n.d.	5.94 ± 0.80	n.d.
*Raq*TA2	n.d.	n.d.	0.06 ± 0.00
*Raq*TA3	n.d.	n.d.	n.d.

n.d. not detected. Reaction conditions: a,b: 5 mM (*R*)-1-phenylethylamine^a^ or (*R*)-1-aminotetralin^b^, 5 mM pyruvate, 0.1 mM PLP in 50 mM KPi, pH 7.5, with cell-free *E. coli* lysate containing different TAs (0.25 mg mL^−1^ of total lysate protein). The increase of 1-phenylethanone or 1-tetralone was measured at 25 °C at 300 nm. c: 5 mM α -ketoglutaric acid, 5 mM d-alanine, 0.5 mM NADH, 40 mU LDH in 50 mM KPi, pH 7.5, with cell-free lysate (0.05–0.0025 mg mL^−1^ total lysate protein), following the depletion of NADH at 340 nm and 25 °C. d: 10 mM α -ketoglutaric acid, 5 mM l-leucine, 0.1 mM PLP, 0.5 mM NAD+, 1 U GDH (l-glutamate dehydrogenase) in 50 mM KPi, pH 8, with cell-free lysate (0.125 mg mL^−1^ total lysate protein), following the formation of NADH at 340 nm and 30 °C. *E. coli* lysate without heterologous transaminase was used as negative control. The reactions were performed in triplicate.

**Table 2:** Conversions of *rac*-1-phenylethylamine, *rac*-1-aminotetralin or *rac*-1-aminoindane with pyruvate catalysed by the candidate enzymes after 24 h at 30 **°**C.

Protein	1-phenylethylamine c [%]^a^ (*e.e.* [%])^b^	1-aminotetralin c [%]^a^ (*e.e.* [%])^b^	1-aminoindane c [%]^a^ (*e.e.* [%])^b^
*Asp*TA1	0	0	0
*Asp*TA2	0	0	0
*Cpu*TA1	38 (60)	48 (91)	48 (93)
*Cpu*TA2	0	0	0
*Cpu*TA3	0	0	0
*Mgi*TA1	50 (>99)	50 (>99)	50 (>99)
*Mgi*TA2	0	4 (4)	3 (9)
*Raq*TA1	0	0	3 (9)
*Raq*TA2	0	0	0
*Raq*TA3	3 (3)	18 (23)	9 (16)
Lysate without TA	0	0	0
No lysate	0	0	0

Reaction conditions: cell-free E. coli lysate containing different TAs (2.0 mg mL^−1^ total lysate protein), 10 mM *rac*-1-phenylethylamine, *rac*-1-aminotetralin or *rac*-1-aminoindane, 20 mM pyruvate, 0.1 mM PLP and 100 mM KPi buffer, pH 7.5, 24 h at 30 °C. a: Conversions determined by rp-HPLC after derivatisation with Marfey’s reagent from the ratio of remaining amine enantiomers; b: e.e. of the remaining (S)-amine.

**Table 3:** Results of transaminase-catalysed amination reactions of pyruvate, α-ketoglutarate, glyoxylic acid, α-keto-γ-(methylthio)-butyrate and phenylpyruvate with *rac*-1-phenylethylamine, propan-2-amine, *rac*-butan-2-amine, cyclohexylamine and dl-alanine after 24 h at 30 °C.

Protein	Amino donor	Pyruvate c [%]^a^	α-Keto- glutarate c [%]^b^	Glyoxylic acid c [%]	α-Keto-γ-(methyl-thio)-butyrate c [%]^b^	Phenyl-pyruvate c [%]^b^
*Cpu*TA1	1-phenylethylamine	40	29	21	23	4
	propan-2-amine	2	1	—	—	—
	butan-2-amine	3	1	—	—	—
	cyclohexylamine	1	1	—	—	—
	dl-alanine	—	56	71	72	67
*Mgi*TA1	1-phenylethylamine	79	74	39	27	2
	propan-2-amine	2	1	—	—	—
	butan-2-amine	2	2	—	—	—
	cyclohexylamine	1	<1	—	—	—
	dl-alanine	—	43	91	59	16
*Raq*TA3	1-phenylethylamine	24	1	4	4	2
	propan-2-amine	2	<1	—	—	—
	butan-2-amine	2	<1	—	—	—
	cyclohexylamine	1	<1	—	—	—
	dl-alanine	—	63	12	17	0

Reaction conditions: cell-free *E. coli* lysate containing different TAs (2.0 mg mL^−1^ total lysate protein), 200 mM *rac*-1-phenylethylamine, *rac*-butan-2-amine or dl-alanine, or 100 mM propan-2-amine or cyclohexylamine, 20 mM pyruvate, α-ketoglutarate, glyoxylic acid, α-keto-γ-(methylthio)-butyrate or phenylpyruvate, 0.1 mM PLP and 100 mM KPi buffer, pH 7.5, 24 h at 30 °C. The conversions were determined by rp-HPLC after derivatisation of the amino acid products with Marfey’s reagent; a: Calculated from the sum of d-Ala und l-Ala for following reason: in case of Ala, substantial racemisation of the initially formed d-Ala by an amino acid racemase present in the cell-free E. coli lysates occurred as checked by incubation of 20 mM d-Ala in the presence of enzyme lysate and PLP at the same concentrations. 34% of l-Ala was formed in the presence of lysate from CpuTA1, respectively MgiTA1 under these conditions. b: Calculated from the amount of d-Glu, d-Met or d-Phe formed as racemisation to the corresponding l-amino acids was found negligible.

**Table 4:** Data-collection, processing and refinement statistics.

Data collection and processing	
Beamline	Elettra XRD1
Wavelength (Å)	0.9717
Unit-cell parameters (Å,°)	154.3, 154.3, 71.0, 90, 90, 120
Space group	P6_2_
Resolution limits (Å)^a^	48.7–2.5 (2.65–2.5)
Total reflections	131076 (19863)
Unique observations	31759 (5191)
Multiplicity	3.9 (3.8)
Completeness (%)	94.8 (96.3)
<I/σI>	5.5 (2.1)
R_merge_	0.211 (0.604)
R_meas_	0.236 (0.679)
CC(1/2)	97.1 (84.3)
Wilson B factor	24.95
Matthews coefficient	3.48
Molecules per asymmetric unit	2
Solvent content (%)	64.7
**Refinement and validation**	
R_work_/R_free_ (%)	16.28/21.81
No. of protein atoms	4451
No. of water molecules	229
No. of ligand atoms	34
R.m.s. deviations	
Bond lengths (Å)	0.007
Bond angles (°)	0.819
Mean B factor	26.6
Ramachandran outliers (%)	0
Most favoured residues (%)	97.9
PDB code	5K3W

^a^Values in parentheses refer to the highest resolution shell.
