# Supplementary material for: Discovery and structural characterisation of new fold type IV-transaminases exemplify the diversity of this enzyme fold
Source: Sci Rep. 2016 Dec 1;6:38183. doi: 10.1038/srep38183 (PMC5131300; doi:10.1038/srep38183)
Supplement: Supplementary Material [file srep38183-s1.pdf]

Supplementary material

Discovery and structural characterisation of new fold type IV-transaminases exemplify the diversity of this enzyme fold

Tea Pavkov-Keller[1], Gernot A. Strohmeier[1], Matthias Diepold[1], Wilco Peeters[2], Natascha Smeets[2], Martin Schürmann[2], Karl Gruber[1,3], Helmut Schwab[1,4], Kerstin Steiner[1]

Supplemental tables

Table S1. Sequence identities (on protein level) of the newly identified proteins to the known (*R*)-ATAs from *Aspergillus terreus* (XP_001209325, AT-ϖTA) and *Arthrobacter* sp. KNK168 (ABN35871).

Protein	AT-ϖTA	ABN35871	aa	MW	pI
*Asp*TA1	26.9%	25.7%	288	31.3	5.25
*Asp*TA2	18.6%	21.3%	281	31.2	6.00
*Cpu*TA1	21.4%	19.0%	305	32.7	4.66
*Cpu*TA2	15.7%	14.2%	376	41.2	5.14
*Cpu*TA3	16.1%	15.7%	264	28.5	5.71
*Mgi*TA1	17.0%	18.5%	294	31.1	5.08
*Mgi*TA2	16.6%	15.5%	372	40.4	5.05
*Raq*TA1	24.9%	25.2%	287	31.7	6.06
*Raq*TA2	24.3%	23.7%	309	34.1	5.8
*Raq*TA3	18.9%	21.2%	265	28.9	6.06

Table S2. Conserved amino acids of Fold class IV PLP-dependent enzymes according to Höhne et al., 2010. Marked in bold are amino acids found in the (*R*)-ATA subfamily. Marked in grey are the amino acids that do not fit the motif of the prediction.

**Protein**	**31**	**36**	**38**	**40**	**95ff**	**105ff**	**prediction[a]**
ADCL	F/Y	F	T	X	zxK	RGY	
D-ATA	F*	Y	V	K(R/X)	zYzQ	RxH*	
BCAT	Y	F	G	R(K)	YzR	zGz	
**R-ATA**	**H/R***	**Y**	**V/T**	**S(T/A/H/P)**	**(F(Y)VE(ANQ)**	**-**	
ABN35871	**H**	**Y**	**V**	**H**	**FVS**		R-ATA
AT-ωTA	**H**	**Y**	**V**	**S**	**FVE**	RGT	R-ATA
*Asp*TA1	F	**Y**	**V**	**P**	VYLQ	RDH	D-ATA
*Asp*TA2	M	F	G	R	YxK	G	BCAT?
*Cpu*TA1	**R**	F	**T**	**A**	LFAK	-	ADCL?, R-ATA?
*Cpu*TA2	Y	F	G	K	YLR	G	BCAT
*Mgi*TA1	**R**	F	S	G	LVIK	-	ADCL?, R-ATA?
*Mgi*TA2	Y	F	G	K	YLR	G	BCAT
*Raq*TA1	F	**Y**	**V**	**A**	**VYLQ**	RDF	D-ATA
*Raq*TA2	Y	F	G	R	YIR	G	BCAT
*Raq*TA3	F	F	**T**	R	K	RGQ	ADCL

[a]R-ATA: (*R*)-aminotransferase, D-ATA: D-amino acid aminotransferase, BCAT: branched chain aminotransferase, ADCL: 4-amino-4-deoxychorismate lyase
*chainB
z represents one of the hydrophobic amino acids valine, leucine, isoleucine, methionine

Table S3. Sequences of primers used for cloning and mutagenesis in this study. Sequences of restriction sites are underlined, start and stop codons are indicated with boxes and mutated bases are highlighted in bold letters.

Primer Name (mutation)	Sequence
AspTA1(Nde)_for	TCACATATGATTCCGGGCGTGCCG
AspTA1(Hind)_rev	TCAAAGCTTTTACAGCGCGGCGATGCG
AspTA2(Nde)_for	TCACATATGGACGCCCTGTTTTGGCAC
AspTA2(Hind)_rev	TCAAAGCTTTTACTTGCGGCCGGTGCTTTC
CpuTA1(Nde)_for	TCACATATGACCCGTGCGACCCTCCTG
CpuTA1(Hind)_rev	TCAAAGCTTTCAGTCGGTGCGGGCGAG
synCpuTA1(Nco)_for	AATCACCATGgCCCGTGCAACCCTGCTG
synCpuTA1(Xho-Stop)_rev	AATCACTCGAGATCGGTACGTGCCAGCAGATATGC
CpuTA2(Nde)_for	TCACATATGACTTCTACCAGCATCTCCCTTACCAG
CpuTA2(Hind)_rev	TCAAAGCTTCTACGCGTCGAGGCGCGTC
CpuTA3(Nde)_for	TCACATATGGACGCGAGCAGCACCCTC
CpuTA3(Hind)_rev	TCAAAGCTTTCAGGCTGCGAGCGGCAG
MgiTA1(Nde)_for	TCACATATGACCTGGCGTTTCGCGC
MgiTA1(Hind)_rev	TCAAAGCTTTCAGTCGCGCGGCGAGAG
MgiTA2(Nde)_for	TCACATATGACTCTCACCGACACGAACGACAC
MgiTA2(Hind)_rev	TCAAAGCTTTCAGTCGTCGAGGCGCAGCATC
RaqTA1(Nde)_for	TCACATATGACCAGAACGGTATACCTTAACGGG
RaqTA1(Hind)_rev	TCAAAGCTTTTACCGGCTCGTCGCTTCCAC
RaqTA3(Nde)_for	TCACATATGTGGATTAATGGTGTGGCGGC
RaqTA3(Hind)_rev	TCAAAGCTTTTAACAGCTCTGGAGCAGGAAATCGC

Table S4. HPLC analysis conditions.

**Instrument:**	Agilent 1100, DAD detector
**Column:**	EC 150/3 Nucleodur C18 Gravity 3 µm (Macherey-Nagel, Düren, Germany)
**Eluent A:**	0.01% formic acid in water
**Eluent B:**	acetonitrile
**Flow rate:**	0.7 mL/min
**Column temperature:**	30°C
**Injection volume:**	1.0 µL
**Detection**	UV, 340 nm

Method A: 0-2.0 min: 40% B; 2.0-7.0 min: 60% B

Method B: 0-2.0 min: 40% B; 2.0-8.5 min: 60% B

Method C: 0-2.0 min: 40% B; 2.0-10.5 min: 50% B

Method D: 0-3.0min: 35% B; 9.0-13.0min: 60% B

Table S5. Retention times of compounds after derivatisation with Marfey's reagent.

**Compound**	**Method**	**t_{ret} [min]** (***S***)	(***R***)
Alanine	A, B, C	1.81	2.12
1-Phenylethylamine	A	6.47	5.90
1-Aminotetralin	B	7.24	7.54
1-Aminoindane	C	9.63	9.94
Glutamic acid	D	1.69	1.86
Glycine	D	2.05	
Alanine	D	2.28	2.96
Methionine	D	3.61	5.72
Phenylalanine	D	6.65	8.25
1-Phenylethylamine	D	11.15	10.37

Supplemental figures

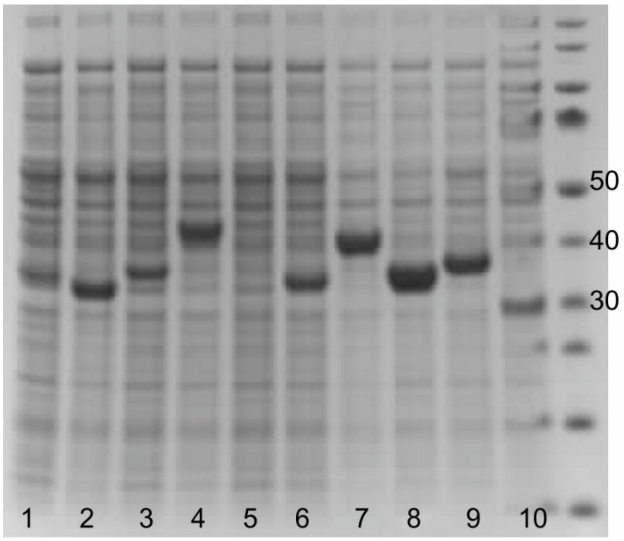

Fig. S1. SDS-PA gel of cell free lysate obtained by sonication and centrifugation of *E. coli* TOP10F’ expressing various putative transaminases (pMS470 vector). 1: *Asp*TA1, 2: *Asp*TA2, 3: *Cpu*TA1, 4: *Cpu*TA2, 5: *Cpu*TA3, 6: *Mgi*TA1, 7: *Mgi*TA2, 8: *Raq*TA1, 9: *Raq*TA2, 10: *Raq*TA3. St: Page Ruler Prestained Protein Ladder (Thermo Scientific). For molecular weights please refer to Table S1.

Chromatograms

Chromatograms to data presented in Table 2

Chrom. S1a. DL-Alanine (method A, B, C)

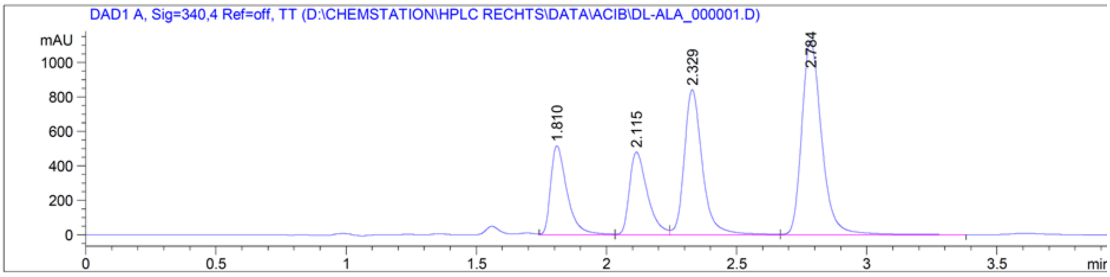

Signal 1: DAD1 A, Sig=340,4 Ref=off, TT

Peak #	RetTime [min]	Type	Width [min]	Area [mAU*s]	Height [mAU]	Area %
1	1.810	VV	0.0661	2256.96753	514.90405	15.4462
2	2.115	VV	0.0727	2290.46802	479.62823	15.6755
3	2.329	VV	0.0744	4068.29102	840.91064	27.8426
4	2.784	VB	0.0813	5996.04492	1123.69043	41.0357

Chrom. S1b. D-Alanine (method A, B, C)

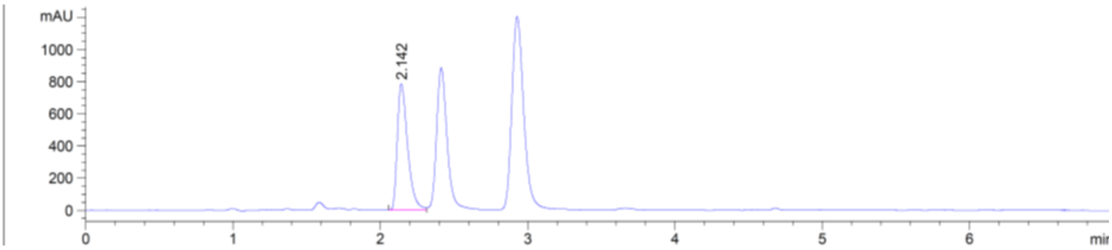

Chrom. S2a. *rac*-1-Phenylethylamine (method A)

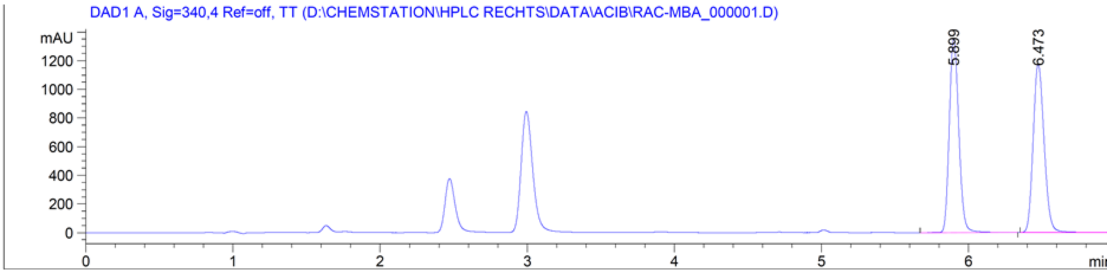

Signal 1: DAD1 A, Sig=340,4 Ref=off, TT

Peak #	RetTime [min]	Type	Width [min]	Area [mAU*s]	Height [mAU]	Area %
1	5.899	BB	0.0705	6094.79492	1353.74890	49.9462
2	6.473	BBA	0.0817	6107.92969	1174.17004	50.0538

Chrom. S2b. (*R*)-1-Phenylethylamine (method A)

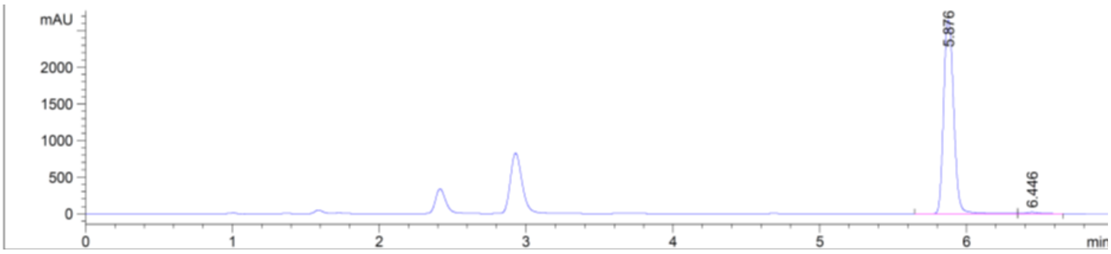

Chrom. S3a. *rac*-1-Aminotetralin (method B)

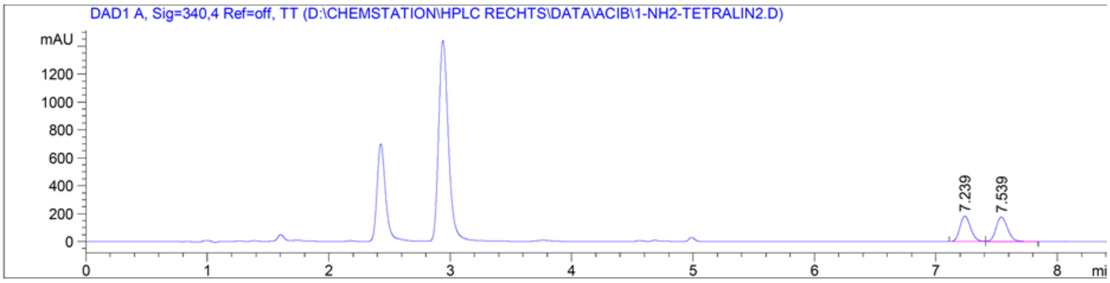

Signal 1: DAD1 A, Sig=340,4 Ref=off, TT

Peak #	RetTime [min]	Type	Width [min]	Area [mAU*s]	Height [mAU]	Area %
1	7.239	BV	0.0953	1115.38049	182.45490	49.1824
2	7.539	VB	0.1026	1152.46472	175.62392	50.8176

Chrom. S3b. (*R*)-1-Aminotetralin (method B)

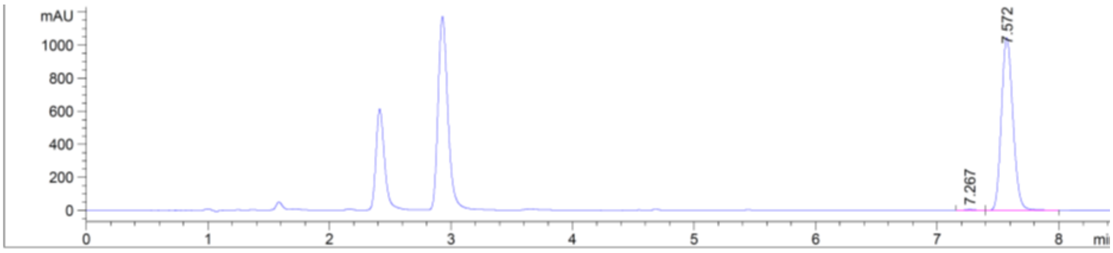

Chrom. S4a. (*S*)-1-Aminoindane + (*R*)-1-aminoindane; (*S*)/(*R*) = 2:1 (method C)

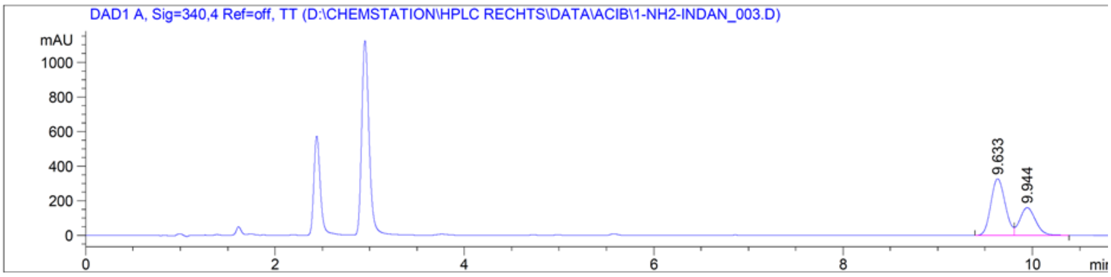

Signal 1: DAD1 A, Sig=340,4 Ref=off, TT

Peak #	RetTime [min]	Type	Width [min]	Area [mAU*s]	Height [mAU]	Area %
1	9.633	BV	0.1653	3428.74829	326.94998	65.0403
2	9.944	VB	0.1777	1842.98218	159.63591	34.9597

Chrom. S4b. (*R*)-1-Aminoindane (method C)

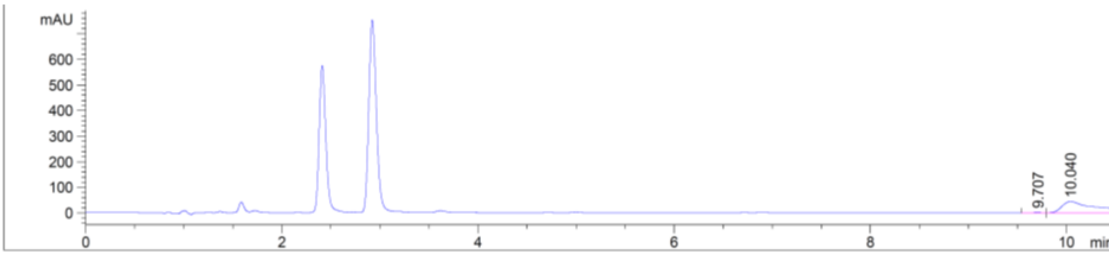

Chrom. S5. Reaction of *rac*-1-phenylethylamine with pyruvate in the presence of *Mgi*TA1 after 24h (method A)

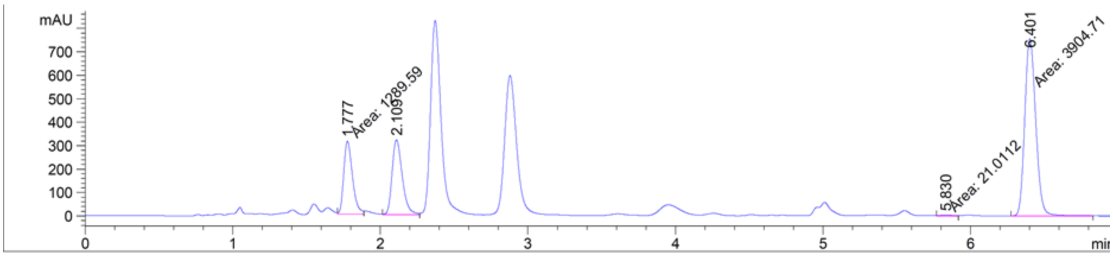


```
Signal 1: DAD1 A, Sig=340,4 Ref=off, TT
```

Peak #	RetTime [min]	Type	Width [min]	Area [mAU*s]	Height [mAU]	Area %
1	1.777	MF	0.0689	1289.59448	311.81287	19.1483
2	2.109	VV	0.0737	1519.44324	318.30035	22.5612
3	5.830	FM	0.0832	21.01121	4.20805	0.3120
4	6.401	FM	0.0861	3904.71362	756.02844	57.9785

Chrom. S6. Reaction of *rac*-1-aminotetralin with pyruvate in the presence of *Mgi*TA1 after 24h (method B)

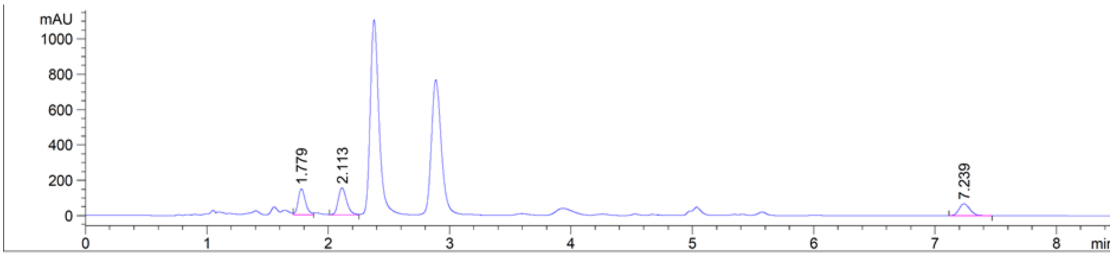


```
Signal 1: DAD1 A, Sig=340,4 Ref=off, TT
```

Peak #	RetTime [min]	Type	Width [min]	Area [mAU*s]	Height [mAU]	Area %
1	1.779	VV	0.0634	606.34552	146.26454	34.8297
2	2.113	VV	0.0736	721.30048	151.44264	41.4330
3	7.239	BB	0.0953	413.23959	67.58504	23.7373

Chrom. S7. Reaction of *rac*-1-aminoindane with pyruvate in the presence of *Mgi*TA1 after 24h (method C)

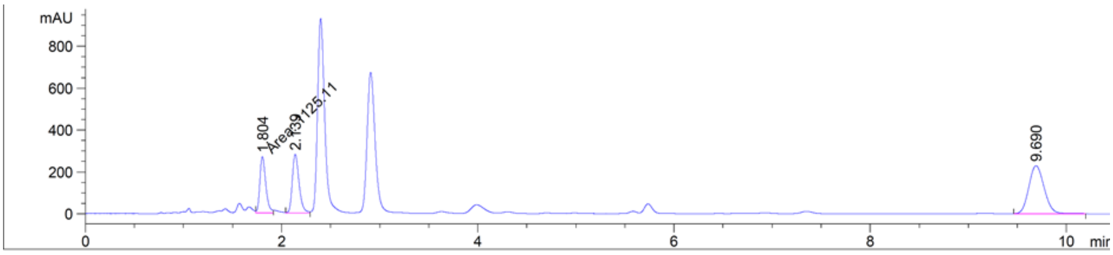

Signal 1: DAD1 A, Sig=340,4 Ref=off, TT

Peak #	RetTime [min]	Type	Width [min]	Area [mAU*s]	Height [mAU]	Area %
1	1.804	MF	0.0700	1125.11365	267.84354	22.8153
2	2.139	VV	0.0736	1328.67896	278.60529	26.9432
3	9.690	BB	0.1692	2477.61401	229.12068	50.2415

Chromatograms to data presented in Table 3

Chrom. S8. Analysis of an amino acid mixture consisting of glycine, DL-alanine, glutamic acid (D/L = 1.77), methionine (D/L = 1.56) and phenylalanine (D/L = 1.95) (method D)

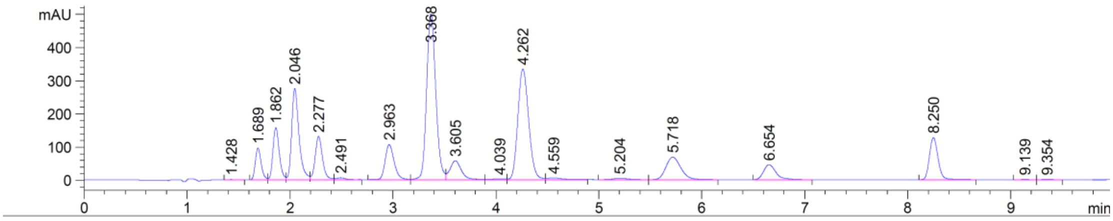

Signal 1: DAD1 A, Sig=340,4 Ref=off, TT

Peak #	RetTime [min]	Type	Width [min]	Area [mAU*s]	Height [mAU]	Area %	
1	1.428	BB	0.0612	5.26829	1.30279	0.0447	
2	1.689	BV	0.0605	382.82028	96.13377	3.2515	L-Glu
3	1.862	VV	0.0668	685.78912	157.63814	5.8248	D-Glu
4	2.046	VV	0.0741	1370.44299	275.18820	11.6399	Gly
5	2.277	VV	0.0765	657.11340	130.98158	5.5812	L-Ala
6	2.491	VB	0.1022	47.20288	6.54390	0.4009	
7	2.963	BV	0.0947	663.15881	106.38354	5.6326	D-Ala
8	3.368	VV	0.0963	3089.62793	498.82541	26.2418	
9	3.605	VV	0.1175	453.25974	57.77902	3.8498	L-Met
10	4.039	VV	0.1335	29.15875	2.99647	0.2477	
11	4.262	VV	0.1156	2451.39453	334.01700	20.8210	
12	4.559	VB	0.1615	65.88130	5.75575	0.5596	
13	5.204	BV	0.1677	51.97421	4.60585	0.4414	
14	5.718	VB	0.1570	695.67987	68.17644	5.9088	D-Met
15	6.654	BB	0.1302	379.61288	45.09370	3.2243	L-Phe
16	8.250	BB	0.0899	736.12823	126.57189	6.2523	D-Phe
17	9.139	BV	0.0902	2.87513	5.22499e-1	0.0244	
18	9.354	VB	0.0868	6.28778	1.13243	0.0534	

Chrom. S9. Reaction of *rac*-1-phenylethylamine with α-keto glutarate in the presence of *Cpu*TA1 after 24h (method D)

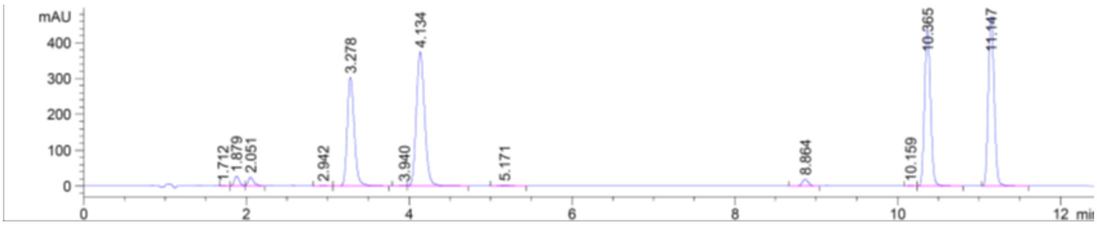

Signal 1: DAD1 A, Sig=340,4 Ref=off, TT

Peak #	RetTime [min]	Type	Width [min]	Area [mAU*s]	Height [mAU]	Area %	
1	1.712	VV	0.0629	7.19441	1.68215	0.0757	
2	1.879	VV	0.0660	119.31408	27.83022	1.2561	D-Glu
3	2.051	VV	0.0744	119.44154	24.27901	1.2575	Gly
11	10.365	VB	0.0823	2249.63672	435.61060	23.6840	
12	11.147	BB	0.0799	2391.17651	473.24359	25.1742	

Chrom. S10. Reaction of *rac*-1-phenylethylamine with glyoxylic acid in the presence of *Cpu*TA1 after 24h (method D)

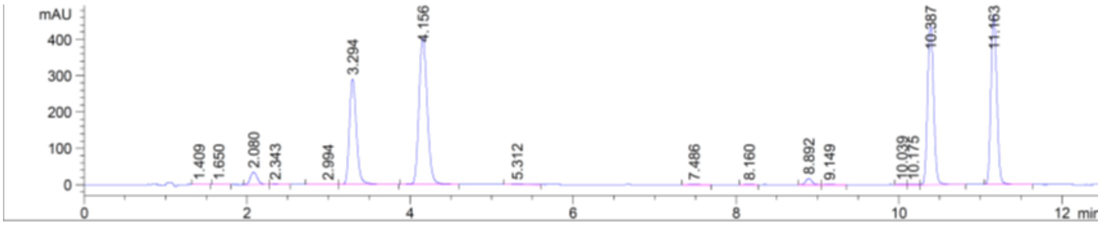

Signal 1: DAD1 A, Sig=340,4 Ref=off, TT

Peak #	RetTime [min]	Type	Width [min]	Area [mAU*s]	Height [mAU]	Area %
1	1.409	BB	0.0695	4.38706	9.21968e-1	0.0457
2	1.650	BB	0.0737	5.73751	1.10215	0.0598
3	2.080	BV	0.0958	202.20061	33.81403	2.1066
15	10.387	VB	0.0809	2268.66943	442.19571	23.6361
16	11.163	BB	0.0797	2363.67725	469.97104	24.6260

Chrom. S11. Reaction of *rac*-1-phenylethylamine with α-keto-γ-(methyl-thio)-butyrate in the presence of *Cpu*TA1 after 24h (method D)

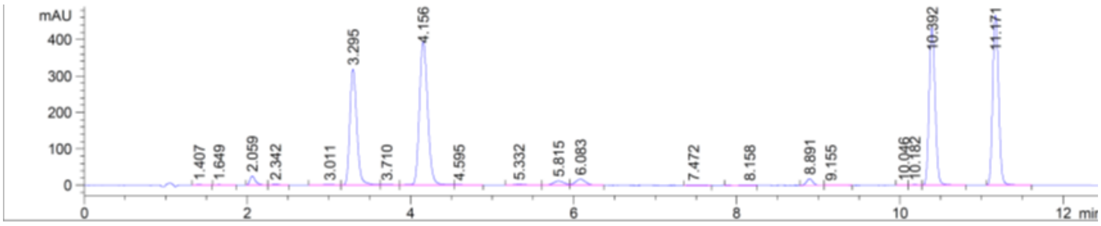

Signal 1: DAD1 A, Sig=340,4 Ref=off, TT

Peak #	RetTime [min]	Type	Width [min]	Area [mAU*s]	Height [mAU]	Area %
11	5.815	VV	0.1472	105.22019	11.24384	1.0698
19	10.392	VB	0.0811	2229.21704	433.05884	22.6650
20	11.171	BB	0.0797	2348.27148	466.37875	23.8754

Chrom. S12. Reaction of *rac*-1-phenylethylamine with phenylpyruvate in the presence of *Cpu*TA1 after 24h (method D)

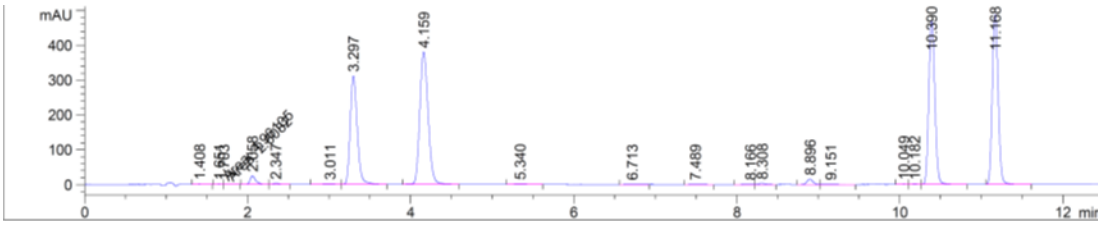

Signal 1: DAD1 A, Sig=340,4 Ref=off, TT

Peak #	RetTime [min]	Type	Width [min]	Area [mAU*s]	Height [mAU]	Area %
13	8.308	VV	0.0927	20.39646	3.36693	0.2082
18	10.390	VB	0.0811	2433.62720	472.60370	24.8421
19	11.168	BB	0.0788	2453.55640	487.07599	25.0456
